# Case Report: foetal gastroschisis with ideal pregnancy outcomes under multidisciplinary treatment management

**DOI:** 10.3389/fped.2024.1358856

**Published:** 2024-02-28

**Authors:** Shuhua Liu, Jingyu Qian, Qiuru Li, Dehong Liu, Bin Zhang, Xianxia Chen

**Affiliations:** ^1^ Department of Obstetrics and Gynecology, Anhui Province Maternity and Child Health Hospital, Hefei, China; ^2^ Department of Obstetrics and Gynecology, Anhui Provincial Women and Children’s Medical Center, Hefei, China; ^3^ Department of Obstetrics and Gynecology, Hefei Maternal and Child Health Hospital, Hefei, China

**Keywords:** gastroschisis, multidisciplinary treatment, management, foetal, abdominal defect

## Abstract

**Background:**

Gastroschisis has increased in recent years, however, complicated gastroschisis is associated with higher mortality, as well as higher health care costs and disease burdens from short- and long-term complications.

**Case introduction:**

A woman aged 25 years old at 37 + 1 weeks gestation (gravida 2; para 0) was admitted to the hospital because of foetal gastroschisis. Targeted quaternary ultrasound performed at our hospital showed that 34 mm of the abdominal wall was interrupted continuously, an intestinal echo with a range of approximately 88 × 50 mm was seen bulging outwards the local area close to the intestinal wall showed a 34 × 23 m anecho, and the foetus was measuring 2 weeks smaller than expected. After MDT including the maternal-foetal medicine, ultrasound, paediatric surgery, neonatal intensive care unit (NICU), and anaesthesiology departments, caesarean section was performed at 37 + 2 weeks. A baby boy was delivered, the small intestine, large intestine and stomach were seen outside of the abdomen, the abdominal cavity was excluded from the defect on the right side of the umbilical cord, the mesentery was shortened, and the intestinal tube had obvious oedema After paediatric surgical discussion, silo bag placement and delayed closure was performed, the placement process was smooth. One week following silo placement, the abdominal contents had been fully reduced below the fascia following daily partial reductions of the viscera,and the second stage of the operation was performed under general anaesthesia. The newborn was successfully discharged from the hospital 20 days after the operation and was followed up, with good growth, normal milk intake and smooth bowel movements.

**Conclusions:**

The diagnosis and treatment of complicated gastroschisis needs to be carried out under multidisciplinary team treatment. Delivery by cesarean section after 37 weeks is feasible.Immediate postpartum surgery is possible, and the choice of surgical modality is determined by the child's condition, emphasizing that it should be performed without adequate sedation under anaesthesia. A standardized postoperative care pathway appropriate to risk should be developed to optimize nutritional support and antibiotic use, and standardized enteral feeding practices should be sought with long-term follow-up.

## Background

Gastroschisis (GS) is a congenital abdominal defect that is usually located on the right side of the umbilical cord, characterized by the discharge of the intraperitoneal tube and other abdominal contents into the amniotic cavity, and ultrasonography reveals that the foetal bowel floats in the amniotic fluid (1–3). GS develops in the early embryonic period, and the cause of most gastroschisis cases is unknown (4–6). Due to the toxic effect of amniotic fluid and the constriction of internal splanchnic blood vessels (7), secondary damage occurs to the foetal abdominal organs when they are immersed in amniotic fluid for a long time. There are two types of GS according to the severity of the disease: the simple type and complex type. Complicated gastroschisis often involves many complications, such as bowel perforation, atresia, volvulus, and even necrosis, which are associated with a poor prognosis of the disease (8, 9), and the probability of foetal death is 7.6 times higher in complex cases than in simple cases (8). As a solitary type of foetal abdominal wall defect, the simple type of GS is characterized by no intestinal complications, accounting for 89% of cases, and the prognosis is relatively good (10).

In a study on birth defects in the Chinese population, the incidence of gastroschisis in the offspring of patients under 20 years of age was 10.62 per 10,000 live births; however, the incidence of gastroschisis in the offspring of parents between the ages of 25 and 29 years was 1.51 per 10,000 live births (11). Additionally, the prevalence of gastroschisis has increased, from 3.6 per 10,000 live births in 1995–2005 to 4.9 per 10,000 live births in 2006–2012 (12). Similarly, in North America, a 16-year retrospective study showed that gastroschisis affects approximately 4 per 10,000 live births, and the prevalence appears to be increasing (13).

Prenatal ultrasound can easily identify gastroschisis in a foetus, with the bowel floating in the amniotic membrane without covering the membrane (5). Neonates with gastroschisis are usually born mildly premature (14), but the overall survival rate for patients with gastroschisis is significant (3, 14) thanks to multidisciplinary treatment (MDT) by the antenatal ultrasound, neonatal intensive care, surgery, quality care, anaesthesiology, and obstetrics departments. However, there is no consensus on the timing of pregnancy termination, the prognosis of the foetus, or the modalities of surgical closure for gastroschisis, resulting in differences in procedures for the diagnosis and treatment of foetal gastroschisis (15, 16).This article provides a comprehensive review of the origin, epidemiology, prenatal diagnosis, postpartum treatment, and subsequent follow-up and prognosis of gastroschisis.

## Case presentation

**Figure 1:**
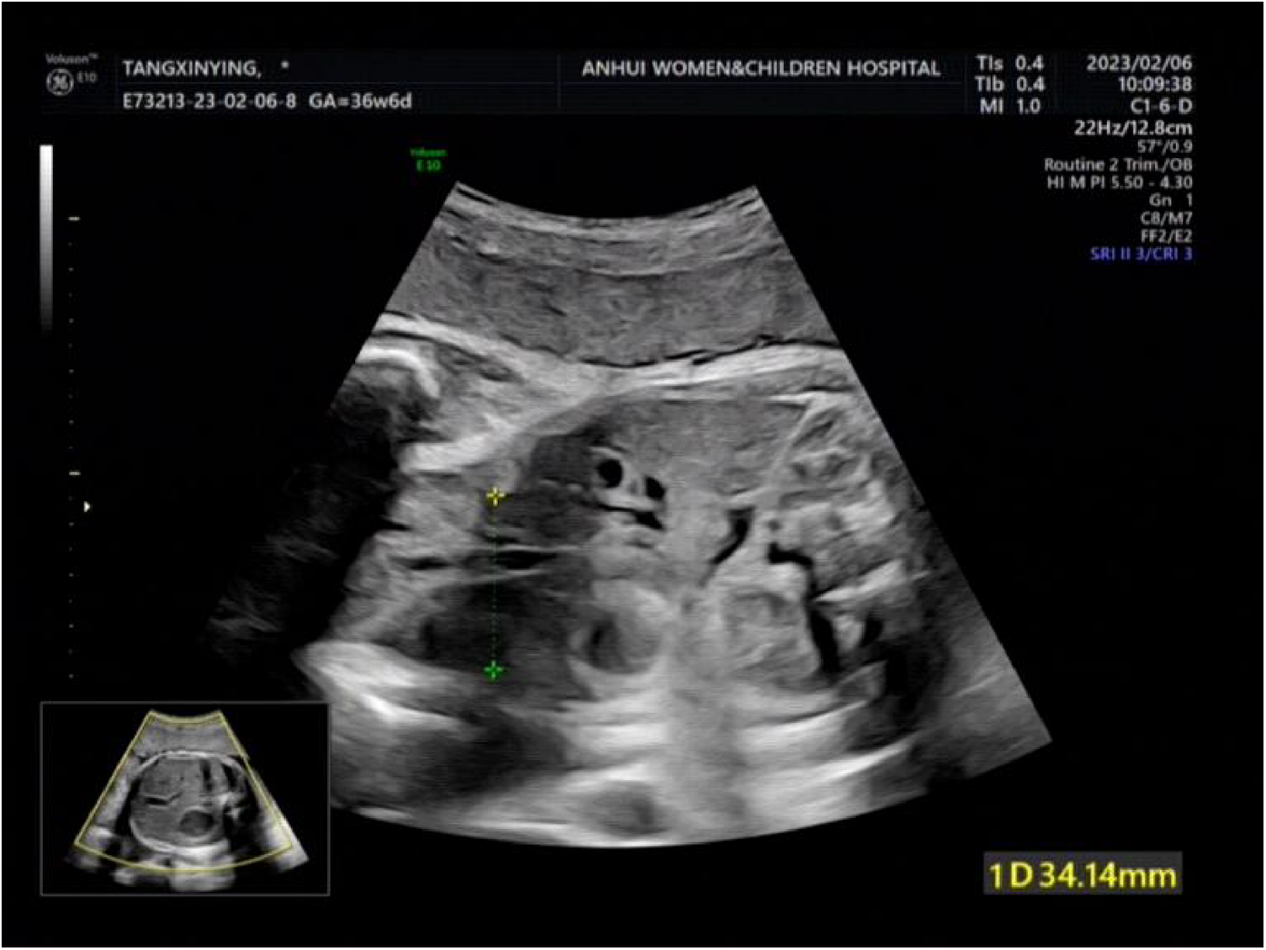
The targeted quaternary ultrasound of our hospital showed that 34 mm abdominal wall defect or abdominal wall continuity was interrupted.

**Figure 2:**
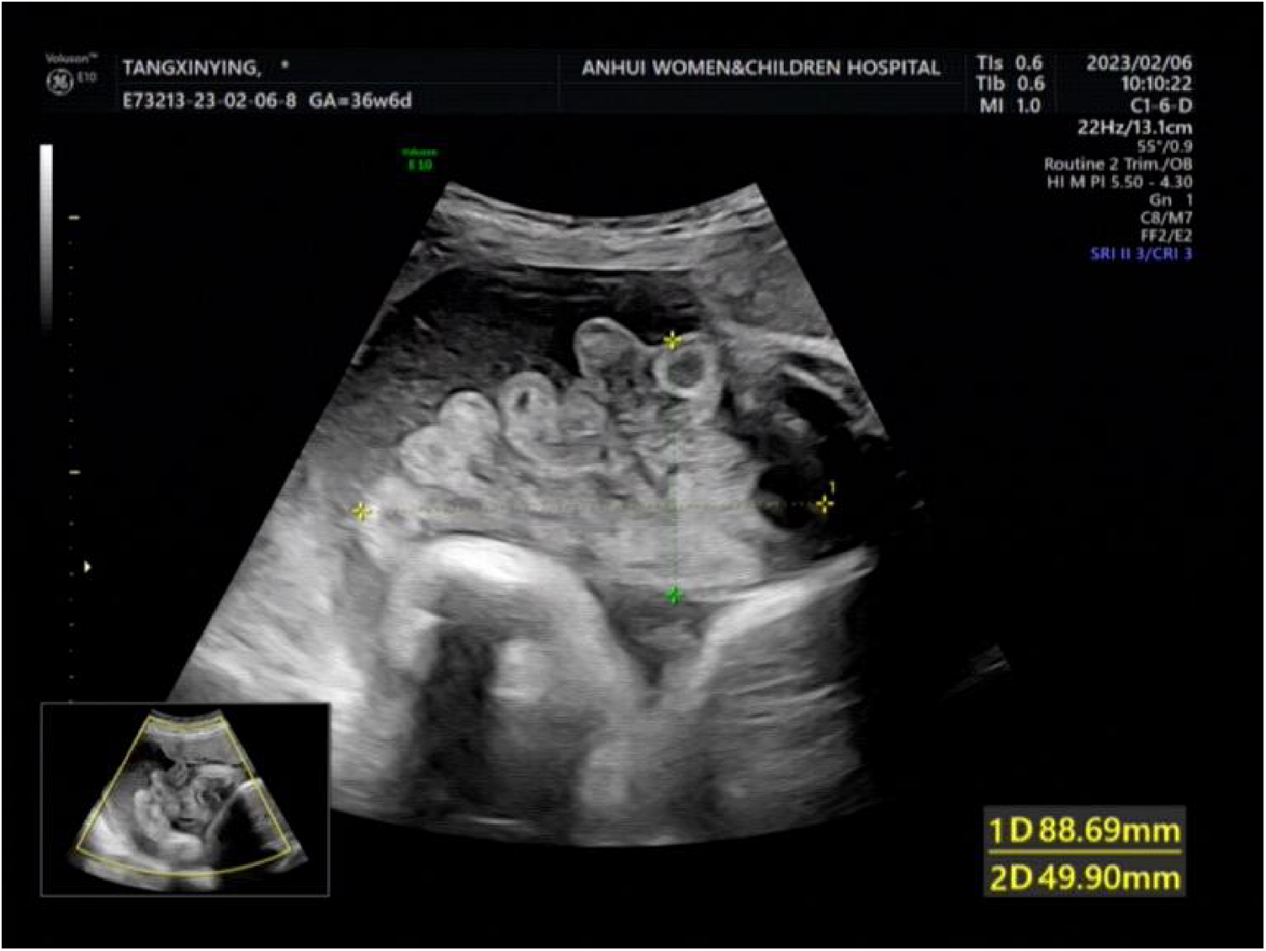
The intestinal echo with a range of about 88 × 50 mm was seen by bulging outward.

**Figure 3:**
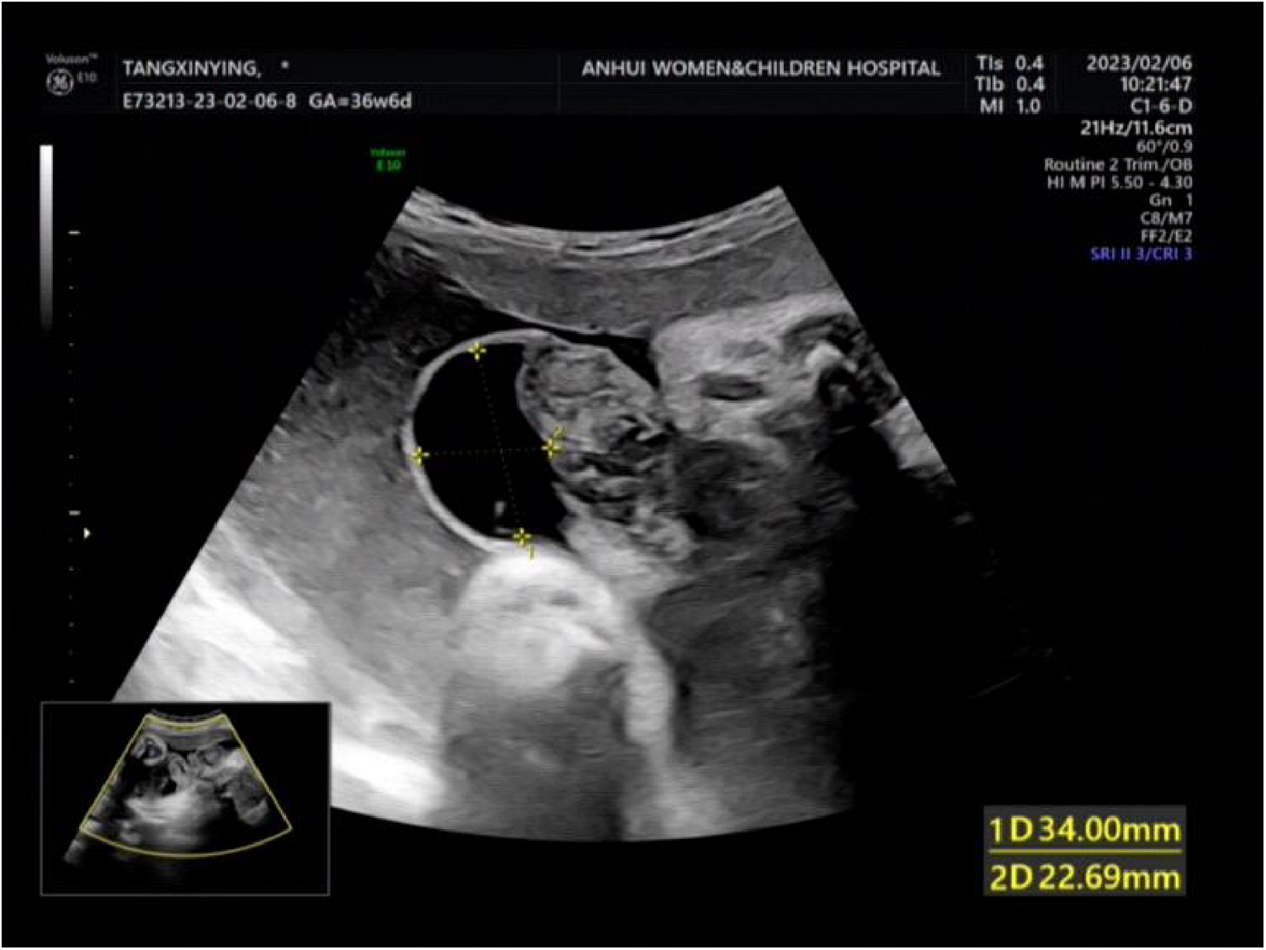
The local close to the intestinal wall showed 34 × 23 m anecho.

**Figure 4:**
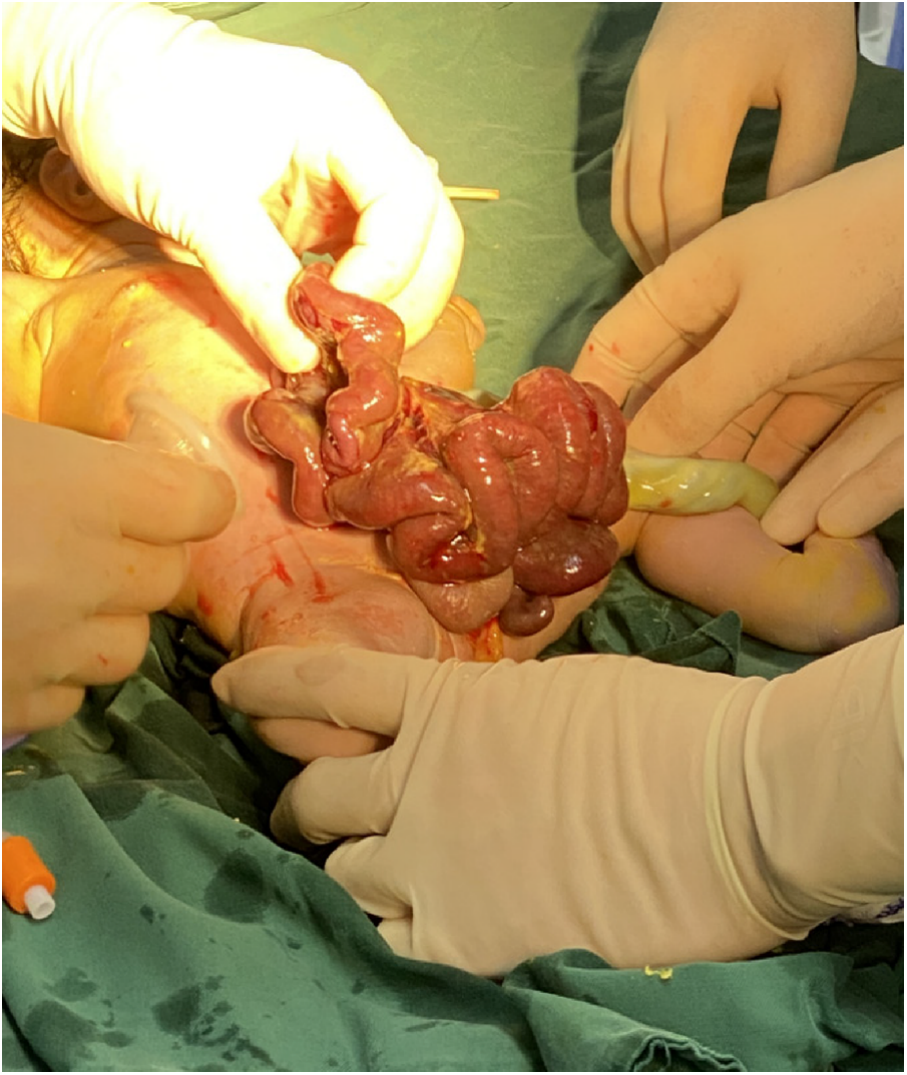
The abdominal cavity was excluded from the defect on the right side of the umbilical cord of the abdomen, and the mesentery was shortened, and the intestinal tube had obvious edema.

**Figure 5:**
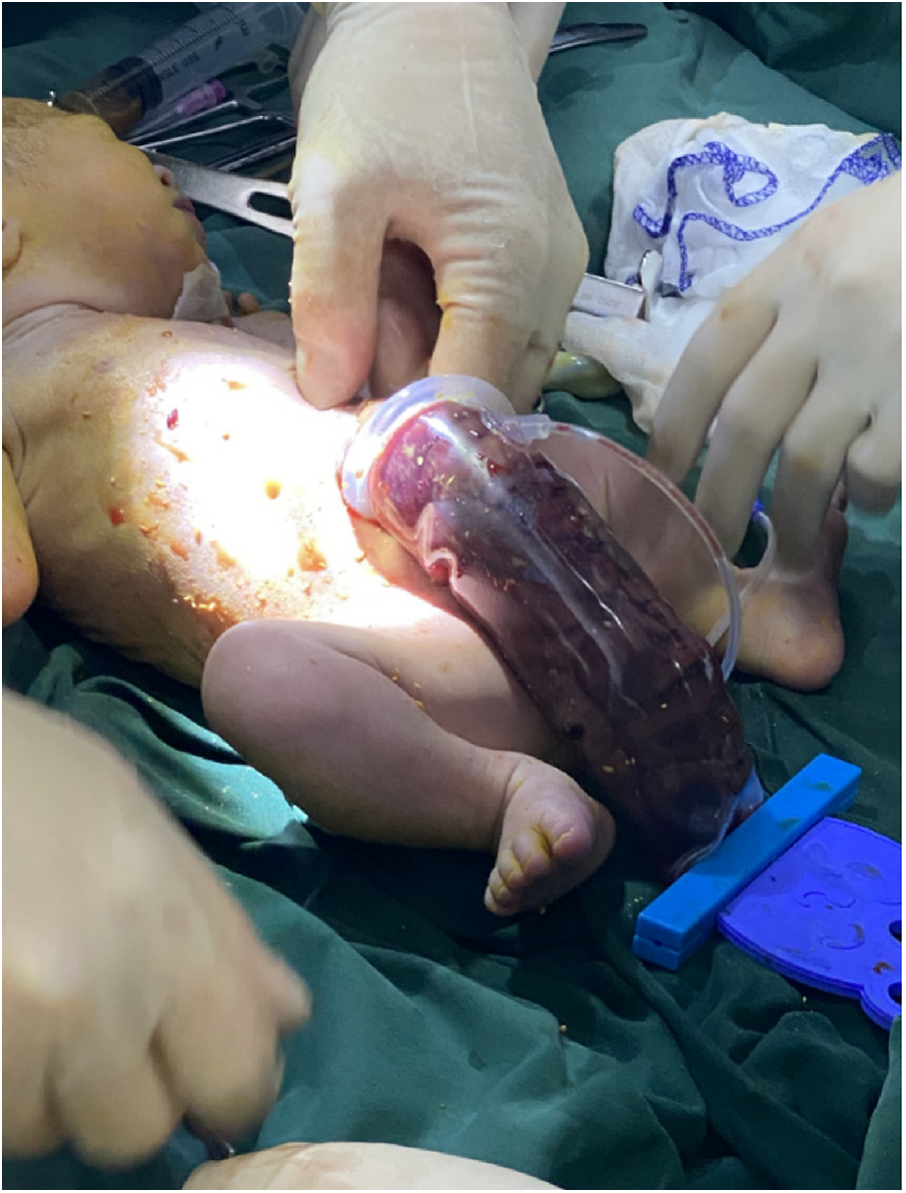
Silo bag placement delayed closure was performed.

**Figure 6:**
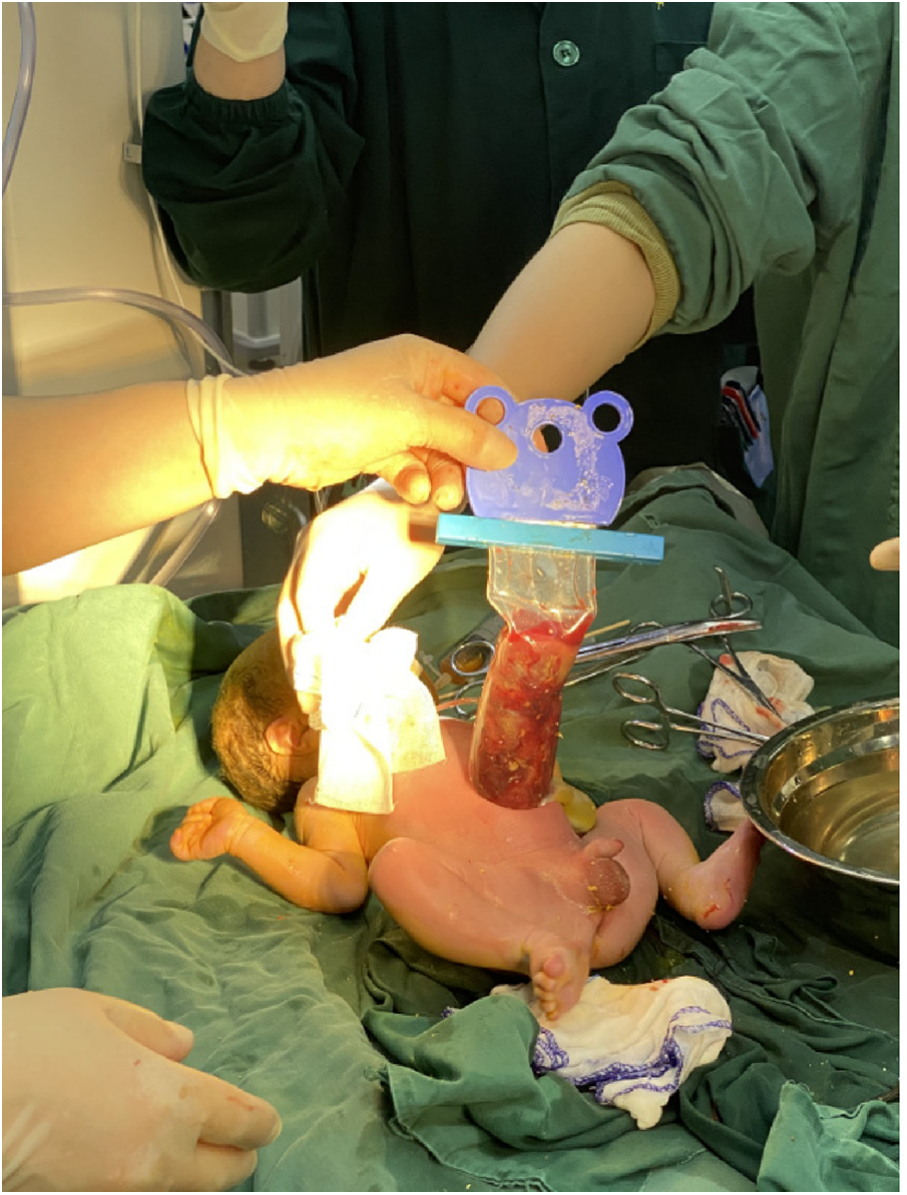
The silo bag is placed.

A woman aged 25 years old at 37 + 1 weeks gestation (gravida 2; para 0) was admitted to the hospital because of foetal gastroschisis. She had regular prenatal examinations during pregnancy and had no history of smoking, or toxic drug use. At 24 + 6 weeks gestation, a four-dimensional ultrasound performed at the local county-level hospital showed that there was an approximately 5-mm wide fissure on the right side of the umbilical cord in the foetal abdomen, and foetal gastroschisis was considered. At 26 weeks gestation, the results of amniotic fluid subchromosomal karyotyping and microarray analysis were normal. Later ultrasound showed that the right navel fissure of the abdomen gradually enlarged, and the bowel was floating in the amniotic fluid. At 36 + 6 weeks gestation, a targeted quaternary ultrasound performed at our hospital showed 34 mm abdominal wall defect or abdominal wall continuity was interrupted (Figure 1), an intestinal echo with a range of approximately 88 × 50 mm was seen bulging outwards (Figure 2), the local area close to the intestinal wall showed a 34 × 23 m anecho (Figure 3), and the foetus was measuring 2 weeks smaller than expected. After MDT including the maternal-foetal medicine, ultrasound, paediatric surgery, neonatal intensive care unit (NICU), and anaesthesiology departments, caesarean section was performed at 37 + 2 weeks. A baby boy was delivered, the small intestine, large intestine and stomach were seen outside of the abdomen, the abdominal cavity was excluded from the defect on the right side of the umbilical cord, the mesentery was shortened, and the intestinal tube had obvious oedema (Figure 4). After paediatric surgical discussion, silo bag placement and delayed closure was performed (Figure 5), the placement process was smooth, and the placement results are shown in Figure 6. One week following silo placement, the abdominal contents had been fully reduced below the fascia following daily partial reductions of the viscera, and the second stage of the operation was performed under general anaesthesia. The surgical methods include intestinal adhesiolysis, appendectomy, and umbiloplasty. The operation process was smooth, the gastric tube was retained after the operation, infection was prevented, and total parenteral nutrition, breast milk and formula milk were added 14 days after the second stage of surgery. The newborn was successfully discharged from the hospital 20 days after the operation and was followed up, with good growth, normal milk intake and smooth bowel movements.

## Discussion and conclusions

### The origin of gastroschisis

Decades ago, gastroschisis was thought to be a common embryonic mechanism associated with defects in the fusion of lateral body folds, causing abnormal closure of the abdominal cavity, similar to other congenital anomalies of the ventral body wall, and was also thought to be a separate malformation of the umbilical cord (17), usually occurring between weeks 4 and 12 after embryogenesis (18, 19). Studies have proposed several possible theories about the origin of gastroschisis, including the following: (1) abnormal fusion of the midline of the asymmetric body fold, thereby preventing the yolk sac from merging into the fixed pedicle, leading to the development of gastroschisis (20). (2) Vascular changes, including weakness and subsequent rupture of the body pleura, and abnormal degeneration of the right umbilical vein (21); in these cases, the vitelline artery ruptures, resulting in infarction and necrosis at the base of the spinal cord (22). In bivascular and thrombotic models, which suggest that the degeneration of the right umbilical vein leaves space for the right umbilical ring, hormonal changes lead to thrombotic damage to adjacent tissues and cause abdominal organ protrusion (23); 3. The rupture of the amniotic membrane at the base of the umbilical cord (24). In 2022, a study by Morris et al. used data from EUROCAT to investigate the prevalence of abnormal vascular rupture and its association with young maternal age, noting that the magnitude of the contribution of vascular destruction to the aetiology is controversial (25). In 1981, scholars first proposed that the rupture of the umbilical mesenteric artery is the underlying mechanism of gastric fissure (22). After decades of research, scholars in 2010 refuted the hypothesis regarding blood vessels (26). The challenge regarding the pathogenesis of gastroschisis involves different hypotheses that have been proposed over the decades. These vessels do not supply the umbilical cord ring and abdominal wall area from an embryological point of view, and if these vessels are abnormal, the survival of the embryo is also problematic (27). Although various theories have reasonable explanations, there is still no consistent view of the origin of gastroschisis.

### Epidemiology of gastroschisis

The incidence of gastroschisis has been inconsistently reported in different regions, with currently reported rates ranging from 3 to 4.5 per 10,000 live births (28). Epidemiological surveys show a brief increase in the incidence of gastroschisis in North and South America and many European countries (29). There are still few studies on gastroschisis in China, and in 2022, a study on the incidence in the Chinese population showed that the incidence of gastroschisis in the offspring of patients under 20 years of age was 10.62 per 10,000 live births; however, the incidence of gastroschisis in the offspring of parents between the ages of 25 and 29 years was 1.51 per 10,000 live births (11). The same findings suggest that the offspring of pregnant adolescents are at high risk of gastroschisis (30, 31). At the same time, there are studies pointing out that racial differences are also factors in the development of gastroschisis; for example, white and Hispanic individuals are more likely to develop foetal gastroschisis (32). However, low-income families with low economic status also have an increased incidence of foetal gastroschisis (33).

### Aetiology of gastroschisis

Looking at studies on the pathogenesis of gastroschisis, most studies were not based on human evidence, which poses a challenging problem and drives the study of risk factors (27). Genetic factors have not been previously reported and have not been clearly established, although cases of gastroschisis among distant relatives have been reported (34, 35). Genetic studies suggest that only 1.2% of children with gastroschisis have chromosomal abnormalities (36). Therefore, the pathogenesis of gastroschisis tends to be related to environmental factors. Studies have shown that the incidence of foetal gastroschisis is associated with maternal smoking during pregnancy (37–39). Similarly, the use of marijuana, cocaine, methamphetamine (39–41), and even depression medications (42) during pregnancy can increase the incidence of gastroschisis. Environmental factors are also high risk factors for the development of gastroschisis, such as the presence of contaminants in the local environment or pesticide abuse (43–45). Some infectious diseases, such as herpes simplex virus and herpes simplex 2, have been associated with the development of gastroschisis (17). In addition to the above factors, the degree of maternal psychological stress may also be involved in the development of foetal malformations (17). A case–control study by Werler et al. evaluating 16 different levels of stress and the risk of gastroschisis found that in the group of cases, the exposure level was 6 times higher than that of the control group, and this study supported the hypothesis that gastroschisis risk factors induce inflammation and oxidative responses (46).

### Prenatal diagnosis of gastroschisis

Accurate prenatal diagnosis of gastroschisis is important, facilitating antenatal physician consultation and subsequent perinatal planning preparation, as well as predicting the intensity of neonatal care and duration of hospitalization. Serum alpha-fetoprotein levels combined with ultrasound can detect 90% of cases of gastroschisis in children,and up to one-quarter of gastroschisis cases can be diagnosed by early nuchal chromosome screening at 14 weeks gestation (14). It is particularly important to distinguish between gastroschisis and omphalocele antenatally because they may be very similar on ultrasonography, but outcomes vary widely between foetal and neonatal outcomes. Omphalocele is caused by an intra-abdominal fold defect and is associated with multisystem abnormalities such as genetic syndromes and the nervous, cardiac, pulmonary, and renal systems. An omphalocele is a defect that occurs within the umbilical ring, and the difference between gastroschisis and omphalocele is the presence of a covered amniotic membrane, the presence of solid internal organs, and the location of the defect relative to cord insertion. Ultrasound in early pregnancy can detect 50% of cases of omphalocele associated with chromosomal abnormalities, as well as other disease syndromes and isolated malformations. The association between gastroschisis and chromosomal abnormalities is not strong, and studies have shown that only 1.2% of children with gastroschisis have chromosomal abnormalities. Therefore, data on birth defects suggest that the 14% abnormality rate may be due to the misdiagnosis of omphalocele, so the possibility of gastroschisis is overestimated (36).

Prenatal diagnosis predicts the severity of intestinal injury at birth, and complicated gastroschisis is associated with adverse neonatal outcomes. Studies have shown that foetal MRI measuring the extra-abdominal-excluded bowel volume can predict the need for silo bag treatment with reasonable accuracy, leading to better prenatal consultation and better surgeon preparation (47, 48).

### Perinatal management of gastroschisis

Prompt intervention can improve perinatal outcomes, and we should develop treatment plans for pregnancy and the neonatal period and complete perinatal management (including pregnancy management, ectopic management, prompt transport, and neonatal surgery). The significance of prenatal multidisciplinary consultation is to regulate the scope of perinatal management and postnatal treatment to improve the survival rate and quality of life of infants. In the traditional model, prenatal consultation for structural foetal malformations is mainly performed by obstetricians, and prognosis assessment and perinatal management may not be comprehensive. The prenatal multidisciplinary consultation model with the participation of all relevant disciplines can provide a comprehensive review, and while completing prenatal consultation, treatment and follow-up plans can be formulated to appropriately address various diseases of foetuses and newborns and follow up on disease outcomes.

There is still no uniform conclusion on the timing of pregnancy termination and the manner of delivery of foetuses with gastroschisis. The goal of therapeutic preterm birth for gastroschisis is to completely detach the foetus from the inflammatory substances of the intrauterine environment to reduce intestinal damage. Studies in animal experiments have shown that increased exposure to amniotic fluid can aggravate damage to the intestinal circulation (49, 50). However, there is no definitive conclusion on whether increased exposure to amniotic fluid in human foetuses will increase intestinal damage, so the effectiveness and safety of early delivery remain controversial. One study suggested no significant difference in the postnatal outcomes (duration of hospital stay, total parenteral nutrition, and days of surgical closure of gastroschisis) of delivery before 34 weeks compared with usual obstetric care (51), and other studies have shown similar results (52, 53). Similarly, there are also studies showing that the severity of intestinal damage actually decreases with gestational age (54). Although there is evidence that the mean gestational age at spontaneous labour of foetuses with gastroschisis is less than 37 weeks (55), researchers believe that planned preterm birth may lead to reduced bowel damage and improved outcomes (56–58). However, the termination of pregnancy before 36 weeks was associated with a significant increase in adverse neonatal outcomes and hospital bills (59–62). Therefore, it is currently recommended that the timing of pregnancy termination in foetuses with gastroschisis without foetal or maternal complications should be after 37 weeks.

### Postpartum management of gastroschisis

Postnatal management of gastroschisis requires close collaboration between multidisciplinary teams and the development of risk-appropriate standardized care pathways, with an overall good prognosis for most infants. We start our review in the following sections.

### Circulating volume management of gastroschisis

The initial goals of treatment are to avoid infusion with umbilical vein vascular access as much as possible to maintain physiological homeostasis, necessary respiratory support, thermal retention, and bowel protection. The peri-intestinal area can be protected by wrapping it in gauze soaked with saline. After the birth of a child with gastroschisis, a nasogastric tube is placed to promote intestinal decompression. To replace the loss of nasogastric tube fluid, 10% glucose sodium chloride solution can be injected to maintain end-organ perfusion, and normal neonatal capacity can be reflected by the child's vital signs, capillary refill, and urine output. Crystalloid (normal saline) or colloids should be given with caution when using volume resuscitation for hypovolemia and metabolic acidosis, and it is now accepted that fluid resuscitation should be retained beyond maintenance requirements when the foetus is hypovolaemic (14). It has been thought that the prolonged immersion of the bowel in amniotic fluid in foetuses with gastroschisis has resulted in fluid loss in the third space, so routine fluid resuscitation after birth is recommended. However, routine fluid resuscitation has been associated with adverse outcomes, including increased mechanical ventilation and hospital stays and an increased incidence of bacteraemia (63).

### Treatment of gastroschisis surgery

At present, surgical treatment is divided into 3 categories, including surgical closure in the first stage of surgery, delayed closure of silo bag placement, and sutureless closure. However, before the advent of the silo bag in the 1990s, surgical primary fascial closure was the main closure method. With the advent of silo bags in the early 20th century, for foetuses with complicated gastroschisis, closure can be delayed by placing silo bags and transferring the foetuses to the NICU to continue treatment. The transparent prefabricated silo bottom with a coil spring reinforced deformable ring (Figure 5) does not require stitching, which means that silos can be placed on a conscious baby. Due to the foetal bowel tube being immersed in amniotic fluid for a long time, oedema may occur, the abdominal wall of the foetus can be outside the abdominal cavity for a long time, the contents of the peritoneum are reduced, and the bowel tube with obvious oedema may not be able to enter the abdominal cavity; this may lead to increased physiological pressure in the abdominal cavity, leading to adverse consequences. Silo bag placement and delayed closure was initially used in cases where closure for gastroschisis could not be performed, and outcomes have been shown to be comparable or better than those of emergency primary closure (47, 48). Delayed closure by silo bag placement was associated with improved outcomes (ventilator days, duration of enteral feeding, and reduction in infection rates); however, when all studies were included, primary surgical closure was associated with improved outcomes (64). The latest advancement in the surgical treatment of gastroschisis is sutureless closure, a technique that shrinks the organs and stretches the umbilical cord through the defect, which stay in place without any fascial sutures (65, 66). In approximately 2 weeks, fascial defects shrink circumferentially to form granulation wounds, epithelialize within 4 weeks, and form a near-normal umbilical cord in appearance. Sutureless closure has been shown to be both the primary closure technique and a delayed treatment strategy (67). Studies have shown that patients with sutureless cord closure have fewer days of ventilation, general anaesthesia, and antibiotic use compared with those with primary suture therapy. Future research may focus on further identifying sutureless closure as a feasible and safe surgical modality for closing these defects (68, 69).

### Antibiotic use

Infectious complications are common during gastroschisis treatment. Antibiotic treatment for gastroschisis includes “prophylaxis” before surgical closure and anti-infective therapy for confirmed or suspected infection. The incidence of surgical site infection varies with suture techniques, with the highest incidence among patients with delayed closure after silo bag placement and the lowest incidence among those with sutureless closure (70). Children with complicated gastroschisis also have a higher rate of surgical site infection and bloodstream infection associated with the central line (71). In a study of 400 children with gastroschisis, the incidence rates of surgical site infections and central line-related bloodstream infections were 13% and 15%, respectively (70). Antibiotic use rates vary widely in children with gastroschisis, so it is important to develop a standardized antibiotic regimen to minimize infection and avoid unnecessary or inappropriate antibiotic therapy (72).

### Nutritional management of gastroschisis

Neonates with gastroschisis require total parenteral nutrition (TPN) at different stages through a peripherally inserted central catheter (PICC). Enteral feeds (preferably breast milk) usually begin approximately 14 days after surgical closure (73). Initiation of enteral nutrition depends on the presence of clinical features (dilation, defecation, and resolution of biliary-nasogastric drainage), and the risks associated with long-term TPN-associated liver disease should be avoided. Enteral feeds are usually initiated by a continuous nasogastric route and given when tolerated (74). The rate of early feeding is what determines the duration of TPN and the duration of hospital stay, with short-term outcomes for isolated gastroschisis including a survival rate close to 100%, a total duration of TPN averaging approximately three weeks, and a duration of stay in the NICU averaging four to five weeks (68). Infants with complicated gastroschisis have a longer dependence on TPN and an increased susceptibility to recurrent sepsis and PNALD (72).

### Outcomes for newborns

Survival outcomes also vary depending on the type of gastroschisis, with overall survival rates well over 90% for simple gastroschisis and neurological development comparable to matched cohort results (75). However, newborns with complicated gastroschisis tends to have a lower median body mass index (BMI) and weight z scores (76). Similarly, another study showed that children with intestinal failure due to complicated gastroschisis were more likely to have cognitive problems at school age than those with simple gastroschisis (77). However, there was no difference in overall quality of life or physical functioning of patients with gastroschisis compared to the general population (77, 78).

### Follow-up and existing problems in the later stage

With the advancement of medical technology, the prognosis of patients with intestinal failure has improved significantly; likewise, the short-term outcomes of complicated gastroschisis have also been significantly improved, and the organ transplant rate (liver, small intestine) and survival rate are also high, but the dependence on family TPN and nasal feeding has also been significantly improved (79). Some patients develop particularly severe inflammatory bowel injury at birth, and additional surgery may be needed later, such as repair of intestinal atresia or stenosis or secondary bowel injury due to volvulus or necrotizing enterocolitis, eventually leading to short bowel syndrome (80), and, as the most common complication, intestinal obstruction (81). Furthermore, long-term follow-up has found that adult patients with gastroschisis are dissatisfied with the appearance of abdominal scars, especially missing navels (78). However, umbilical preserving operations have long since become standard (82).

## Conclusion

Gastroschisis is a common neonatal congenital malformation in obstetrics. Reports of gastroschisis have increased in recent years. Its treatment requires close cooperation between multidisciplinary teams, such as maternal-foetal medicine, ultrasound medicine, neonatal intensive care, paediatric surgery, nursing, and anaesthesia departments, in prenatal consultation, intrapartum and postoperative care. There is controversy regarding the mode of delivery and the timing of pregnancy termination, and with successful teamwork and treatment, we recommend caesarean delivery at term (37 weeks). Immediate postpartum surgery is possible, and the choice of surgical modality is determined by the child's condition, emphasizing that either silo placement or sutureless closure can be performed without the need for general anesthesia. A standardized postoperative care pathway appropriate to risk should be developed to optimize nutritional support and antibiotic use, and standardized enteral feeding practices should be sought with long-term follow-up.

## Data Availability

The raw data supporting the conclusions of this article will be made available by the authors, without undue reservation.

## References

[1] Aktoz F Ozyuncu O Tanacan A Fadiloglu E Unal C Soyer T Gestational outcomes of pregnancies with prenatally detected gastroschisis and omphalocele. Fetal Pediatr Pathol. (2019) 38(4):282–9. 10.1080/15513815.2019.1585501 30892123

[2] Haddock C Skarsgard ED. Understanding gastroschisis and its clinical management: where are we? Expert Rev Gastroenterol Hepatol. (2018) 12(4):405–15. 10.1080/17474124.2018.1438890 29419329

[3] Willborg BE Ibirogba ER Trad ATA Sbragia L Potter D Ruano R. Is there a role for fetal interventions in gastroschisis management? an updated comprehensive review. Prenat Diagn. (2021) 41(1):159–76. 10.1002/pd.5820 32876346

[4] CDC. Prevention. Facts About Gastroschisis. Division of Birth Defects and Developmental Disabilities. Atlanta, GA: Centers for Disease Control and Prevention (2019).

[5] Ferreira RG Mendonça CR Gonçalves Ramos LL de Abreu Tacon FS Naves do Amaral W Ruano R. Gastroschisis: a systematic review of diagnosis, prognosis and treatment. J Matern Fetal Neonatal Med. (2022) 35(25):6199–212. 10.1080/14767058.2021.1909563 33899664

[6] South AP Stutey KM Meinzen-Derr J. Metaanalysis of the prevalence of intrauterine fetal death in gastroschisis. Am J Obstet Gynecol. (2013) 209:114.e1–13. 10.1016/j.ajog.2013.04.032 23628262

[7] Langer JC Longaker MT Crombleholme TM Bond SJ Finkbeiner WE Rudolph CA Etiology of intestinal damage in gastroschisis. I: effects of amniotic fluid exposure and bowel constriction in a fetal lamb model. J Pediatr Surg. (1989) 24(10):992–7. 10.1016/s0022-3468(89)80200-3 2530329

[8] Bergholz R Boettcher M Reinshagen K Wenke K. Complex gastroschisis is a different entity to simple gastroschisis affecting morbidity and mortality-a systematic review and meta-analysis. J Pediatr Surg. (2014) 49(10):1527–32. 10.1016/j.jpedsurg.2014.08.001 25280661

[9] Arnold MA Chang DC Nabaweesi R Colombani PM Bathurst MA Mon KS Risk stratification of 4344 patients with gastroschisis into simple and complex categories. J Pediatr Surg. (2007) 42(9):1520–5. 10.1016/j.jpedsurg.2007.04.032 17848242

[10] Nitzsche K Fitze G Rüdiger M Birdir C. Prenatal prediction of outcome by fetal gastroschisis in a tertiary referral center. Diagnostics. (2020) 10(8):540. 10.3390/diagnostics10080540 32751744 PMC7460378

[11] Chen X Lou H Chen L Muhuza MPU Chen D Zhang X. Epidemiology of birth defects in teenage pregnancies: based on provincial surveillance system in eastern China. Front Public Health. (2022) 10:1008028. 10.3389/fpubh.2022.1008028 36561870 PMC9763884

[12] Jones AM Isenburg J Salemi JL Arnold KE Mai CT Aggarwal D Increasing prevalence of gastroschisis-14 states, 1995–2012. MMWR Morb Mortal Wkly Rep. (2016) 65:23–6. 10.15585/mmwr.mm6502a2 26796490

[13] Baerg J Kaban G Tonita J Pahwa P Reid D. Gastroschisis: a sixteen-year review. J Pediatr Surg. (2003) 38(5):771–4. 10.1016/jpsu.2003.50164 12720191

[14] Skarsgard ED. Management of gastroschisis. Curr Opin Pediatr. (2016) 28(3):363–9. 10.1097/MOP.0000000000000336 26974976

[15] Landisch RM Yin Z Christensen M Szabo A Wagner AJ. Outcomes of gastroschisis early delivery: a systematic review and meta-analysis. J Pediatr Surg. (2017) 52(12):1962–71. 10.1016/j.jpedsurg.2017.08.068 28947324

[16] Allin BS Tse WH Marven S Johnson PR Knight M. Challenges of improving the evidence base in smaller surgical specialties, as highlighted by a systematic review of gastroschisis management. PLoS One. (2015) 10(1):e0116908. 10.1371/journal.pone.0116908 25621838 PMC4306505

[17] Chuaire Noack L. New clues to understand gastroschisis. Embryology, pathogenesis and epidemiology. Colomb Med. (2021) 52(3):e4004227. 10.25100/cm.v52i3.4227 PMC8973314 35431359

[18] Sadler TW. The embryologic origin of ventral body wall defects. Semin Pediatr Surg. (2010) 19(3):209–14. 10.1053/j.sempedsurg.2010.03.006 20610194

[19] Sadler TW Feldkamp ML. The embryology of body wall closure: relevance to gastroschisis and other ventral body wall defects. Am J Med Genet C Semin Med Genet. (2008) 148C(3):180–5. 10.1002/ajmg.c.30176 18655098

[20] Feldkamp ML Carey JC Sadler TW. Development of gastroschisis: review of hypotheses, a novel hypothesis, and implications for research. Am J Med Genet A. (2007) 143A(7):639–52. 10.1002/ajmg.a.31578 17230493

[21] deVries PA. The pathogenesis of gastroschisis and omphalocele. J Pediatr Surg. (1980) 15(3):245–51. 10.1016/s0022-3468(80)80130-8 6445962

[22] Hoyme HE Higginbottom MC Jones KL. The vascular pathogenesis of gastroschisis: intrauterine interruption of the omphalomesenteric artery. J Pediatr. (1981) 98(2):228–31. 10.1016/s0022-3476(81)80640-3 6450826

[23] Lubinsky M. A vascular and thrombotic model of gastroschisis. Am J Med Genet A. (2014) 164A(4):915–7. 10.1002/ajmg.a.36370 24458365

[24] Shaw A. The myth of gastroschisis. J Pediatr Surg. (1975) 10(2):235–44. 10.1016/0022-3468(75)90285-7 123582

[25] Morris JK Wellesley D Limb E Bergman JEH Kinsner-Ovaskainen A Addor MC Prevalence of vascular disruption anomalies and association with young maternal age: a EUROCAT study to compare the United Kingdom with other European countries. Birth Defects Res. (2022) 114(20):1417–26. 10.1002/bdr2.2122 36369770 PMC10099853

[26] Sadler TW Rasmussen SA. Examining the evidence for vascular pathogenesis of selected birth defects. Am J Med Genet A. (2010) 152A(10):2426–36. 10.1002/ajmg.a.33636 20815034

[27] Feldkamp ML Carey JC. The pathogenesis of gastroschisis. Birth Defects Res. (2023) 115(5):515–6. 10.1002/bdr2.2140 36541834

[28] Kilby MD. The incidence of gastroschisis. Br Med J. (2006) 332:250–1. 10.1136/bmj.332.7536.250 16455699 PMC1360382

[29] Loane M Dolk H Bradbury I. EUROCAT working group. Increasing prevalence of gastroschisis in Europe 1980–2002: a phenomenon restricted to younger mothers? Paediatr Perinat Epidemiol. (2007) 21(4):363–9. 10.1111/j.1365-3016.2007.00820.x 17564594

[30] Castilla EE Mastroiacovo P Orioli IM. Gastroschisis: international epidemiology and public health perspectives. Am J Med Genet C Semin Med Genet. (2008) 148C:162–79. 10.1002/ajmg.c.30181 18655097

[31] Salemi JL Pierre M Tanner JP Kornosky JL Hauser KW Kirby RS Maternal nativity as a risk factor for gastroschisis: a population-based study. Birth Defects Res A Clin Mol Teratol. (2009) 85:890–6. 10.1002/bdra.20612 19645051

[32] Williams LJ Kucik JE Alverson CJ Olney RS Correa A. Epidemiology of gastroschisis in metropolitan Atlanta, 1968 through 2000. Birth Defects Res A Clin Mol Teratol. (2005) 73(3):177–83. 10.1002/bdra.20114 15744732

[33] Torfs CP Velie EM Oechsli FW Bateson TF Curry CJ. A population-based study of gastroschisis: demographic, pregnancy, and lifestyle risk factors. Teratology. (1994) 50(1):44–53. 10.1002/tera.1420500107 7974254

[34] Beaudoin S. Insights into the etiology and embryology of gastroschisis. Semin Pediatr Surg. (2018) 27(5):283–8. 10.1053/j.sempedsurg.2018.08.005 30413258

[35] Binet A Ceaux E Bory JP Poli-Merol ML François-Fiquet C. Récidive de grossesse avec fœtus atteint de laparoschisis: à propos d'un cas [recurrence of pregnancies with gastroschisis: a case report]. Arch Pediatr. (2015) 22(10):1039–41. 10.1016/j.arcped.2015.07.011 26382639

[36] Mastroiacovo P Lisi A Castilla EE Martínez-Frías ML Bermejo E Marengo L Gastroschisis and associated defects: an international study. Am J Med Genet A. (2007) 143A:660–71. 10.1002/ajmg.a.31607 17357116

[37] Haddow JE Palomaki GE Holman MS. Young maternal age and smoking during pregnancy as risk factors for gastroschisis. Teratology. (1993) 47:225–8. 10.1002/tera.1420470306 8475465

[38] Feldkamp ML Alder SC Carey JC. A case control population-based study investigating smoking as a risk factor for gastroschisis in Utah, 1997–2005. Birth Defects Res A Clin Mol Teratol. (2008) 82:768–75. 10.1002/bdra.20519 18985693

[39] Draper ES Rankin J Tonks AM Abrams KR Field DJ Clarke M Recreational drug use: a major risk factor for gastroschisis? Am J Epidemiol. (2008) 167:485–91. 10.1093/aje/kwm335 18063593

[40] Weinsheimer RL Yanchar NL. Canadian pediatric surgical network. Impact of maternal substance abuse and smoking on children with gastroschisis. J Pediatr Surg. (2008) 43:879–83. 10.1016/j.jpedsurg.2007.12.032 18485958

[41] Brindle ME Flageole H Wales PW. Canadian pediatric surgery network (CAPSNet). influence of maternal factors on health outcomes in gastroschisis: a Canadian population-based study. Neonatology. (2012) 102:45–52. 10.1159/000336564 22507959

[42] Skarsgard ED Meaney C Bassil K Brindle M Arbour L Moineddin R. Maternal risk factors for gastroschisis in Canada. Birth Defects Res A Clin Mol Teratol. (2015) 103:111–8. 10.1002/bdra.23349 25684659

[43] Root ED Meyer RE Emch ME. Evidence of localized clustering of gastroschisis births in North Carolina, 1999–2004. Soc Sci Med. (2009) 68:1361–7. 10.1016/j.socscimed.2009.01.034 19231056

[44] Waller SA Paul K Peterson SE Hitti JE. Agricultural-related chemical exposures, season of conception, and risk of gastroschisis in Washington state. Am J Obstet Gynecol. (2010) 202:241.e1–6. 10.1016/j.ajog.2010.01.023 20207240

[45] Bassil KL Yang J Arbour L Moineddin R Brindle ME Hazell E Spatial variability of gastroschisis in Canada, 2006–2011: an exploratory analysis. Can J Public Health. (2016) 107(1):e62–7. 10.17269/cjph.107.5084 27348112 PMC6972289

[46] Werler MM Guéry E Waller DK Parker SE. Gastroschisis and cumulative stressor exposures. Epidemiology. (2018) 29(5):721–8. 10.1097/EDE.0000000000000860 29863532 PMC11748026

[47] Pastor AC Phillips JD Fenton SJ Meyers RL Lamm AW Raval MV Routine use of a SILASTIC spring-loaded silo for infants with gastroschisis: a multicenter randomized controlled trial. J Pediatr Surg. (2008) 43(10):1807–12. 10.1016/j.jpedsurg.2008.04.003 18926212

[48] Schlatter M Norris K Uitvlugt N DeCou J Connors R. Improved outcomes in the treatment of gastroschisis using a preformed silo and delayed repair approach. J Pediatr Surg. (2003) 38(3):459–64. 10.1053/jpsu.2003.50079 12632367

[49] Franchi-Teixeira AR Weber Guimarães Barreto M Nogueira B Bittencourt D Violin L Sbragia L. Aminiotic fluid and intrauterine growth restriction in a gastroschisis fetal rat model. Fetal Diagn Ther. (2005) 20(6):494–7. 10.1159/000088037 16260881

[50] Sbragia L Schmidt AF Moraes S Bittencourt DG Gonçalves FL Pereira LA Inflammatory response in a rat model of gastroschisis is associated with an increase of NF-kappaB. Braz J Med Biol Res. (2010) 43(2):160–5. 10.1590/s0100-879(2010005000005 20098844

[51] Shamshirsaz AA Lee TC Hair AB Erfani H Espinoza J Shamshirsaz AA Elective delivery at 34 weeks vs routine obstetric care in fetal gastroschisis: randomized controlled trial. Ultrasound Obstet Gynecol. (2020) 55(1):15–9. 10.1002/uog.21871 31503365

[52] Logghe HL Mason GC Thornton JG Stringer MD. A randomized controlled trial of elective preterm delivery of fetuses with gastroschisis. J Pediatr Surg. (2005) 40(11):1726–31. 10.1016/j.jpedsurg.2005.07.047 16291160

[53] Girsen AI Davis AS Hintz SR Fluharty E Sherwin K Trepman P Effects of gestational age at delivery and type of labor on neonatal outcomes among infants with gastroschisis. J Matern Fetal Neonatal Med. (2021) 34(13):2041–6. 10.1080/14767058.2019.1656191 31409162

[54] Youssef F Laberge JM Baird RJ, Canadian Pediatric Surgery N. The correlation between the time spent in utero and the severity of bowel matting in newborns with gastroschisis. J Pediatr Surg. (2015) 50(5):755–9. 10.1016/j.jpedsurg.2015.02.030 25783374

[55] Lausman AY Langer JC Tai M Seaward PG Windrim RC Kelly EN Gastroschisis: what is the average gestational age of spontaneous delivery? J Pediatr Surg. (2007) 42(11):1816–21. 10.1016/j.jpedsurg.2007.07.005 18022429

[56] Mesas Burgos C Svenningsson A Vejde JH Granholm T Conner P. Outcomes in infants with prenatally diagnosed gastroschisis and planned preterm delivery. Pediatr Surg Int. (2015) 31(11):1047–53. 10.1007/s00383-015-3795-8 26399421

[57] Gelas T Gorduza D Devonec S Gaucherand P Downham E Claris O Scheduled preterm delivery for gastroschisis improves postoperative outcome. Pediatr Surg Int. (2008) 24(9):1023–9. 10.1007/s00383-008-2204-y 18668252

[58] Hadidi A Subotic U Goeppl M Waag KL. Early elective cesarean delivery before 36 weeks vs late spontaneous delivery in infants with gastroschisis. J Pediatr Surg. (2008) 43(7):1342–46. 10.1016/j.jpedsurg.2007.12.050 18639693

[59] Overcash RT DeUgarte DA Stephenson ML Gutkin RM Norton ME Parmar S Factors associated with gastroschisis outcomes. Obstet Gynecol. (2014) 124(3):551–7. 10.1097/AOG.0000000000000425 25162255 PMC4147679

[60] Al-Kaff A MacDonald SC Kent N Burrows J Skarsgard ED Hutcheon JA. Delivery planning for pregnancies with gastroschisis: findings from a prospective national registry. Am J Obstet Gynecol. (2015) 213(4):557:e1–8. 10.1016/j.ajog.2015.06.048 26116872

[61] Harper LM Goetzinger KR Biggio JR Macones GA. Timing of elective delivery in gastroschisis: a decision and cost-effectiveness analysis. Ultrasound Obstet Gynecol. (2015) 46(2):227–32. 10.1002/uog.14721 25377308 PMC4861040

[62] Cain MA Salemi JL Paul Tanner J Mogos MF Kirby RS Whiteman VE Perinatal outcomes and hospital costs in gastroschisis based on gestational age at delivery. Obstet Gynecol. (2014) 124(3):543–50. 10.1097/AOG.0000000000000427 25162254

[63] Jansen LA Safavi A Lin Y MacNab YC Skarsgard ED. Preclosure fluid resuscitation influences outcome in gastroschisis. Am J Perinatol. (2012) 29:307–12. 10.1055/s-0031-1295639 22094919

[64] Kunz SN Tieder JS Whitlock K Jackson JC Avansino JR. Primary fascial closure versus staged closure with silo in patients with gastroschisis: a meta-analysis. J Pediatr Surg. (2013) 48(4):845–57. 10.1016/j.jpedsurg.2013.01.020 23583145 PMC4103994

[65] Bianchi A Dickson AP. Elective delayed reduction and no anesthesia: minimal intervention management for gastroschisis. J Pediatr Surg. (1998) 33:1338–40. 10.1016/s0022-3468(98)90002-1 9766348

[66] Sandler A Lawrence J Meehan J Phearman L Soper R. A “plastic” sutureless abdominal wall closure in gastroschisis. J Pediatr Surg. (2004) 39:738–41. 10.1016/j.jpedsurg.2004.01.040 15137009

[67] Emami CN Youssef F Baird RJ Laberge JM Skarsgard ED Puligandla PS. A risk-stratified comparison of fascial versus flap closure techniques on the early outcomes of infants with gastroschisis. J Pediatr Surg. (2015) 50(1):102–6. 10.1016/j.jpedsurg.2014.10.009 25598103

[68] Fraser JD Deans KJ Fallat ME Helmrath MA Kabre R Leys CM. Sutureless vs sutured abdominal wall closure for gastroschisis: operative characteristics and early outcomes from the midwest pediatric surgery consortium. J Pediatr Surg. (2020) 55(11):2284–8. 10.1016/j.jpedsurg.2020.02.017 32151403

[69] Witt RG Zobel M Padilla B Lee H MacKenzie TC Vu L. Evaluation of clinical outcomes of sutureless vs sutured closure techniques in gastroschisis repair. JAMA Surg. (2019) 154(1):33–9. 10.1001/jamasurg.2018.3216 30325977 PMC6439850

[70] Baird R Puligandla P Skarsgard E Laberge JM. Infectious complications in the management of gastroschisis. Pediatr Surg Int. (2012) 28(4):399–404. 10.1007/s00383-011-3038-6 22159577

[71] Youssef F Gorgy A Arbash G Puligandla PS Baird RJ. Flap versus fascial closure for gastroschisis: a systematic review and meta-analysis. J Pediatr Surg. (2016) 51(5):718–25. 10.1016/j.jpedsurg.2016.02.010 26970850

[72] Al Maawali A Skarsgard ED. The medical and surgical management of gastroschisis. Early Hum Dev. (2021) 162:105459. 10.1016/j.earlhumdev.2021.105459 34511287

[73] Aljahdali A Mohajerani N Skarsgard ED, Canadian Pediatric Surgery Network (CAPSNet). Effect of timing of enteral feeding on outcome in gastroschisis. J Pediatr Surg. (2013) 48(5):971–6. 10.1016/j.jpedsurg.2013.02.014 23701769

[74] Jadcherla SR Gupta A Stoner E Fernandez S Caniano D Rudolph CD. Neuromotor markers of esophageal motility in feeding intolerant infants with gastroschisis. J Pediatr Gastroenterol Nutr. (2008) 47:158–64. 10.1097/MPG.0b013e318162082f 18664867 PMC13455178

[75] van Manen M Hendson L Wiley M Evans M Taghaddos S Dinu I. Early childhood outcomes of infants born with gastroschisis. J Pediatr Surg. (2013) 48(8):1682–7. 10.1016/j.jpedsurg.2013.01.021 23932607

[76] Harris EL Minutillo C Hart S Warner TM Ravikumara M Nathan EA The long term physical consequences of gastroschisis. J Pediatr Surg. (2014) 49(10):1466–70. 10.1016/j.jpedsurg.2014.03.008 25280647

[77] Hijkoop A Rietman AB Wijnen RMH Tibboel D Cohen-Overbeek TE van Rosmalen J. Gastroschisis at school age: what do parents report? Eur J Pediatr. (2019) 178(9):1405–12. 10.1007/s00431-019-03417-5 31325028 PMC6694033

[78] Koivusalo A Lindahl H Rintala RJ. Morbidity and quality of life in adult patients with a congenital abdominal wall defect: a questionnaire survey. J Pediatr Surg. (2002) 37:1594–601. 10.1053/jpsu.2002.36191 12407546

[79] Emil S Canvasser N Chen T Friedrich E Su W. Contemporary 2-year outcomes of complex gastroschisis. J Pediatr Surg. (2012) 47:1521–8. 10.1016/j.jpedsurg.2011.12.023 22901911

[80] Iyer KR. Surgical management of short bowel syndrome. JPEN J Parenter Enteral Nutr. (2014) 38(1 Suppl):53S–9S. 10.1177/0148607114529446 24668996

[81] Friedmacher F Hock A Castellani C Avian A Höllwarth ME. Gastroschisis-related complications requiring further surgical interventions. Pediatr Surg Int. (2014) 30:615–20. 10.1007/s00383-014-3500-3 24736970

[82] Taher H Khalil H Ahmed S Gad M Elezaby B Magdy A Umbilical hernia repair post umbilical cord graft closure of gastroschisis: a cohort study. Int J Surg Case Rep. (2022) 95:107175. 10.1016/j.ijscr.2022.107175 35580418 PMC9117534

